# Iliac Vein Compression Syndrome in an Active and Healthy Young Female

**DOI:** 10.1155/2012/786876

**Published:** 2012-02-14

**Authors:** Sonia Cerquozzi, Graham F. Pineo, Jason K. Wong, Karen A. Valentine

**Affiliations:** ^1^Department of Medicine, Foothills Medical Hospital, Calgary, AB, Canada T2N 2T9; ^2^Department of Radiology, Foothills Medical Hospital, Calgary, AB, Canada T2N 2T9

## Abstract

Iliac vein compression syndrome is a condition involving external compression of the left common iliac vein by the right iliac artery, which was first described in the 1850s. It predominates in females typically between the third and fourth decade of life and has been associated with thrombophilias. Importantly, the syndrome is amenable to endovascular treatment. Here, we describe a case of a young athletic female with an incidental finding of a left iliac vein thrombosis while taking oral contraceptives, who was identified as having iliac vein compression syndrome on follow-up MR venography with positive testing for Factor V Leiden mutation.

## 1. Introduction

May and Thurner first described this anatomic variation in 1956 whereby the left common iliac vein is compressed by the right iliac artery anteriorly and the lumbar vertebral body posteriorly, resulting in lower extremity venous outflow obstruction. Iliac vein compression syndrome (IVCS), also called May-Thurner syndrome and Cockett syndrome, can present as an acute or chronic DVT with unilateral leg pain, edema, and varicosities. Treatment has involved various surgical therapies; however, recently less invasive endovascular strategies have become mainstay treatment. Here, we present a case of a young active female with a new diagnosis of iliac vein compression syndrome and heterozygous Factor V Leiden mutation treated by intravascular stent placement.

## 2. Case Report

A 35-year-old white female who was an elite endurance athlete training for triathlons had a 3-year history of chronic left hip and groin pain which was subsequently investigated by MRI study. Her MRI of the hip and femur incidentally showed distention of the left common and external iliac veins with associated deep vein thrombosis.

At the time, she had no symptoms of leg pain or swelling and had no personal or family history of prior thrombotic events. She had a history of prolonged flights; however, none were taken immediately prior to her investigation. She was on oral contraceptives, which were subsequently discontinued, with no additional medications. She was a lifetime nonsmoker and had no symptoms to suggest concurrent pulmonary embolism. She had a prior hand surgery with no perioperative complications. Her examination was normal with no evidence of leg swelling or asymmetry and no evidence of postthrombotic syndrome.

She was started on anticoagulation and 3 weeks later had an ultrasound of her left lower limb that showed no evidence of deep vein thrombosis, which included the common femoral and external iliac veins. It was concluded that it was difficult to exclude a clot proximal to the iliac vein thus, she was continued on warfarin for 6 months. Following her anticoagulation, she underwent a MR angiogram and venogram. There was no evidence of deep vein thrombosis; however, there was significant narrowing of the upper aspect of the left common iliac vein just as it passed under the right common iliac artery prior to its joining with the right iliac vein. There was no associated dilation of the peripheral left iliac veins, and remaining veins were unremarkable for compression or malformation. These findings were highly suggestive of iliac vein compression. A thrombophilia screen was done following anticoagulation therapy, which showed the patient was heterozygous for Factor V Leiden mutation.

**Figure 1:**
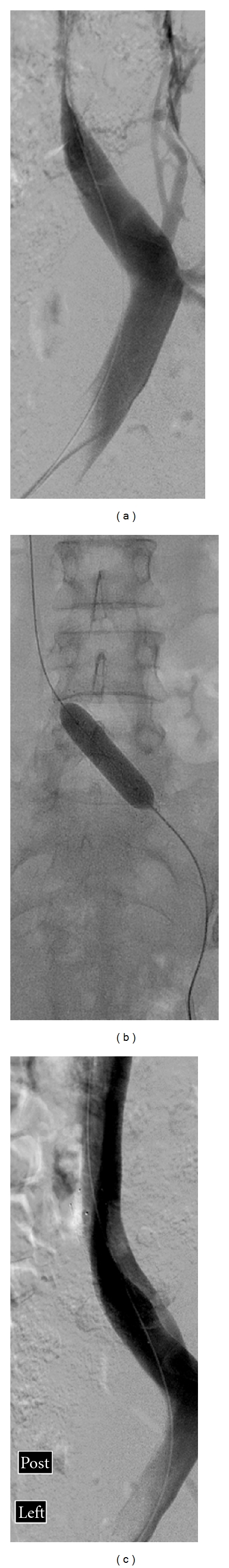
Venogram showing compression of the left common iliac vein (a), and inflation of balloon to expand stent in the left common iliac vein (b) with completion of the venogram after stent placement showing normal flow through left common iliac vein (c).

Soon afterward, the patient had a pelvic venogram illustrating stenosis of the left common iliac vein consistent with external compression with some collateral veins. Pressure measurements were done across the stenosis with a mean pressure gradient of 9 mm Hg. A 14 mm × 60 mm Nitinol self-expanding stent was deployed across the stenosis and dilated with a 12 mm balloon (Figure 1). Good restoration was confirmed on angiography with a mean pressure gradient of 2 mm Hg. She was given 300 mg of Plavix at the time of the procedure and continued Plavix 75 mg for a total of two months. She did well after treatment and continues to take part in her regular training with no further complications.

## 3. Discussion

Iliac vein compression syndrome (IVCS) is characterized by left common iliac vein compression by the right common iliac artery against the fifth lumbar vertebra [1]. Virchow first described IVCS in 1851 whereby he showed a left-sided predominance of iliofemoral DVTs [2]. In 1908, McMurrich, examined 107 cadavers and found adhesions in the iliac veins of 35 cadavers, 32 involving the left common iliac vein [3]. Later, Ehrich and Krumbhaar examined 412 specimens in autopsy in 1943 and found obstructive lesions of collagen and elastin at the mouth of the left common iliac vein [4]. By 1957, May and Thurner examined 430 cadavers, illustrating a decreased venous flow secondary to intimal changes in the left common iliac vein which they described as “spurs” that occurred from chronic compression of the left common iliac vein [1]. In 1965, Cockett and Thomas performed the first clinical trials with 57 patients who presented with acute left iliofemoral DVT from iliac vein compression and subsequently named the syndrome [5].

The pathogenesis is likely a combination of mechanical compression and arterial pulsations on the trapped vein that leads to intimal hypertrophy within the wall of the vessel causing endothelial changes and thrombus formation within the vein [1, 6].

In 1995, Verhaeghe suggested a link between IVCS and genetic factors causing thrombophilia [7]. De Bast and Dahin presented three cases of IVCS associated to Factor V Leiden mutations [8]. Kiernan et al. showed a statistically higher rate of thrombophilia among patients with IVCS and an association with cryptogenic stroke and patent foramen ovales. Among 30 IVCS patients, 12 patients had abnormal thrombophilia screens including prothrombin gene mutations, Factor V Leiden, anticardiolipin antibodies, antiphospholipid syndrome, and Protein C and S deficiency [6].

Oral contraceptives have not been directly evaluated in patients with IVCS although it is well established that they increase the risk of VTE. A 3 to 11 net-fold-increased risk of DVT exists in women on OCPs compared to the general population, and the presence of a hypercoagulable disorder enhances this risk. Patients with Factor V Leiden have an increased baseline risk 7 times higher than noncarriers that can be increased by a factor of 35 if using OCPs [9, 10]. As seen in our case, multiple risk factors were cumulative in contributing to our patient's presentation.

The prevalence of IVCS is approximately 20% [1] with a mean age of 40 years and a female predominance [11]. Interestingly, some authors estimate a 49 to 62% prevalence of this unusual anatomy in patients diagnosed with a DVT [12, 13]. Left sided compression occurs 3 to 8 times more commonly than that on the right side and is generally more asymptomatic. In a case series of 50 people undergoing abdominal CT scans for other medical reasons, approximately a quarter of the patients had greater than 50% left iliac vein compression whereas 2/3 of the patients had over 25% compression. All these patients were asymptomatic and that raises the question whether this is a common anatomical variant [14].

IVCS has numerous variants described in the literature including compression of the left common iliac vein by a tortuous left common iliac artery [15, 16] or by the left hypogastric artery [15, 17]. There is also compression of the right common iliac vein by the right common iliac artery or by the right hypogastric artery [17, 18] or cases of left external iliac vein compression by the left external iliac artery and compression of the right external iliac vein by the right external iliac artery [17]. Rarer cases of compression of the inferior vena cava by the right common iliac artery secondary to high aortic bifurcation [19] and right common iliac vein compression by the left common iliac artery have also been published [18].

Patients typically present with acute edema of the lower limb, most often after surgery, pregnancy, travel, or prolonged immobility. In chronic cases, symptoms caused by venous hypertension occur including claudication, lower extremity pain, swelling, varicose veins, and chronic venous stasis changes such as ulcerations [5]. Asymptomatic cases occur in 15–30% of the population [1]. Physical examination may reveal swelling and diameter of the associated lower limb, varicose veins, hyperpigmentation, or dusky discoloration of the skin, telangiectasia, or ulcerations.

Although the standard test for diagnosing DVT is color duplex imaging, it is not sensitive enough to detect nonocclusive thrombus or intraluminal defects within the common iliac vein and it is inadequate for diagnosis. Imaging of the common iliac vein is difficult and special request for IVC and iliac vein duplex ultrasounds can be ordered but at least 20% of studies are nondiagnostic [20, 21]. CT of the abdomen and pelvis can rule out extrinsic compression as well as acute DVT but a normal study does not rule out IVCS since normal CT cuts are 10 mm and can miss details such as intimal spurs and fibrosis. Chung et al. showed that spiral CT venography for diagnosis of IVCS was useful 75% of the time [22]. MR venography is the imaging of choice and easily depicts areas of compression or obstruction and can localize the presence of collaterals [20, 22, 23]. Intravascular ultrasound can also be used and estimates vessel size and internal wall morphology [23]. Contrast venography is the gold standard and helps with planning therapeutic interventions [18, 20, 21, 23]. Direct pressure measurements can be performed during venography. A difference between the two iliac veins of 2 mmHg at rest or 3 mmHg with exercise is highly suggestive of IVCS. Likewise, evidence of an exaggerated pressure response to exercise signifies the presence of obstruction [1].

Many patients with iliac vein compression clinically improve with endovascular procedures. Noninvasive therapies such as compression stockings have little benefit due to the proximal nature of the compression, and sole use of oral anticoagulation is not adequate. Untreated iliac vein obstruction prevents vein recanalization in 70–80% of patients, and in up to 40% of cases continued clot propagation occurs with further symptoms arising due to chronic clot burden [24, 25]. Thrombolysis or mechanical thrombectomy is treatment options with catheter-directed thrombolysis being best for fresh clots, whereas thrombectomy is in the setting of chronic clot burden [20]. In the past, surgical methods were widely available such as venovenous bypass procedures, mobilization of the right common iliac artery away from the left iliac vein or vein patch angioplasty using a segment of the cephalic vein, with repositioning of the right iliac artery to the retrocaval location [26].

Endovascular treatments such as percutaneous angioplasty with stent placement is favored and was first described in 1995 [27]. Stents are believed to obliterate spur formation and expand the iliac vein back to normal caliber to maintain patency [24]. Some stents are self-expanding while others require balloon inflation [20]. Venous spurs that cause marked luminal narrowing of the common iliac vein are found in approximately 20% of asymptomatic adults and 50% of adults with left iliac venous thrombosis. Rethrombosis rates were lower in patients who received stent placement for their venous spurs than those with spurs left untreated (13% versus 72%). Kwak et al. studied 16 IVCS patients who had metallic stent placements following thrombectomy and showed a 95% and 100% primary and secondary patency rates at 2-year followup [28]. Interestingly, a case of a young women with Factor V Leiden and IVCS treated by endovascular stenting experienced recurrent thrombosis requiring multiple 4 stent placements with persistent symptoms despite collateral blood flow [26]. A review of the literature, compiling six studies with at least 5 IVCS patients treated with endovascular therapy, including catheter-directed thrombolysis with subsequent stent placement found a mean technical success rate of 95% and 1-year patency of 96%, with all patients on anticoagulation after procedure [29].

Although transluminal stenting is first choice, surgical interventions are recognized as appropriate for active females of childbearing age. VTE is one of the leading causes of death among pregnant women in developed countries with the incidence of DVT being 5 times more common in pregnancy [30]. Hartung et al. studied 6 women who had pregnancies following insertion of self-expanding metallic stents, of which, 3 had IVCS. During pregnancy, all women had worsening limb edema with stent or external iliac vein compression occurring in 57% of pregnancies. All women received LMWH, without DVT occurrences and had no evidence of stenosis on duplex scans after partum nor was there evidence of stent structural damage [31]. Current recommendations for young women are to avoid pregnancy within the first year of stent placement given the highest rate of in-stent stenosis [32]. In young women with stents, an ultrasound prior to pregnancy is recommended to verify stent patency. During pregnancy, a repeat scan is suggested at 3, 6, and 8 months and 1 month afterpartum. Thromboprophylaxis with LMWH is recommended from the 3rd month of pregnancy until 1 month postpartum but is more controversial in the setting of stents for iliac vein compression syndrome [31].

## 4. Conclusion

We have reported a presentation of iliac vein compression syndrome in an otherwise, healthy, young women who was treated with a self-expanding stent and continues to remain asymptomatic without complications. Further clinical trials may outline whether alternative approaches are necessary for women of childbearing age. Overall, this vascular anomaly is underrecognized and should remain as a differential diagnosis in the evaluation of left leg deep vein thrombosis and chronic leg edema, especially, in the younger patient population with refractory symptoms or known thrombophilias.
